# A Role of a Newly Identified Isomerase From *Yarrowia lipolytica* in Erythritol Catabolism

**DOI:** 10.3389/fmicb.2018.01122

**Published:** 2018-05-30

**Authors:** Aleksandra M. Mirończuk, Anna Biegalska, Karolina Zugaj, Dorota A. Rzechonek, Adam Dobrowolski

**Affiliations:** Department of Biotechnology and Food Microbiology, Wrocław University of Environmental and Life Sciences, Wrocław, Poland

**Keywords:** erythritol, *Yarrowia lipolytica*, erythritol catabolic pathway, isomerase, glycerol

## Abstract

Erythritol is a natural sweetener produced by microorganisms as an osmoprotectant. It belongs to the group of polyols and it can be utilized by the oleaginous yeast *Yarrowia lipolytica*. Despite the recent identification of the transcription factor of erythritol utilization (*EUF1*), the metabolic pathway of erythritol catabolism remains unknown. In this study we identified a new gene, *YALI0F01628g*, involved in erythritol assimilation. *In silico* analysis showed that *YALI0F01628g* is a putative isomerase and it is localized in the same region as *EUF1*. qRT-PCR analysis of *Y. lipolytica* showed a significant increase in *YALI0F01628*g expression during growth on erythritol and after overexpression of *EUF1*. Moreover, the deletion strain *ΔF01628* showed significantly impaired erythritol assimilation, whereas synthesis of erythritol remained unchanged. The results showed that *YALI0F1628g* is involved in erythritol assimilation; thus we named the gene *EYI1*. Moreover, we suggest the metabolic pathway of erythritol assimilation in yeast *Y. lipolytica*.

## Introduction

**FIGURE 1:**
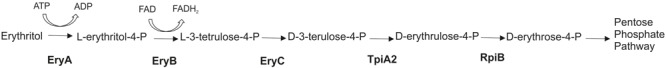
Erythritol catabolism in *Brucella abortus* by Barbier et al. (2014). EryA- erythritol kinase, EryB- erythritol dehydrogenase, EryC- tetrulose-4-phosphate racemase, TpiA2- D-3-tetrulose-4-phosphate isomerase (EryH), RpiB- D-erythrose-4-phosphate isomerase (EryI).

A four-carbon sweet alcohol, erythritol is produced by many microorganisms as an osmoprotectant but also as a storage material in fruits. Erythritol is almost non-caloric, and due to its chemical structure it cannot change the level of the insulin in the blood, so it is safe for diabetics. It has a very low energy level (0–0.2 kcal g^-1^) compared to sucrose (4 kcal g^-1^) or to other sugar alcohols, yielding approximately 2 kcal g^-1^. It does not cause gastrointestinal side effects, since its maximum no-effect dose for causing diarrhea is the highest among polyols. On the industrial scale it is produced by biotechnological fermentation of *Moniliella* spp. or *Candida magnoliae* from glucose (Rzechonek et al., 2018). For many years it was believed that in humans erythritol is excreted in urine within 24 h after consumption (Moon et al., 2010) and that human cells cannot synthesize erythritol. However, the recent study of Hootman et al. (2017) showed that erythritol can be produced by human cells from glucose via the pentose-phosphate pathway (PPP) and about 5–10% of the consumed erythritol is oxidized through the pathway to erythronate. This discovery raised the importance of finding the precise pathway of erythritol synthesis but also its catabolism in eukaryotic cells. Erythritol is an atypical carbon source for microorganisms, although it is a preferential substrate for *Brucella*, a common zoonotic bacterial pathogen, where erythritol metabolism requires the conversion into D-Erythrose 4-phosphate by five enzymatic steps (Barbier et al., 2014; **Figure 1**). Erythritol can also be utilized by the yeast *Yarrowia lipolytica*. This non-conventional, dimorphic, non-pathogenic yeast is able to utilize unconventional carbon sources such as alkanes, polyols, or fatty acids (Groenewald et al., 2014; Mirończuk et al., 2015; Dobrowolski et al., 2016). Because of these unique features it has been studied for many years. *Y. lipolytica* is a model organism for the study of fatty acid synthesis (Papanikolaou et al., 2002; Bellou et al., 2016) and is known for its capacity for production of organic acids (Kamzolova and Morgunov, 2017) and polyols (Rakicka et al., 2017a). Though not unique amongst yeast (Kurtzman et al., 2011) one of the features of this yeast is the capability for erythritol assimilation; thus this microorganism could represent a model organism for study of erythritol catabolism in eukaryotic cells.

In recent studies a few genes involved in erythritol assimilation in *Y. lipolytica* have been identified, but the full pathway remains unknown (Carly and Fickers, 2018; Regnat et al., 2018). Hitherto the known elements of the catabolic machinery are the transcriptional factor *EUF1* (YALI0F01562g) (Rzechonek et al., 2017), erythrulose kinase *EYK1* (YALI0F01606g) (Carly et al., 2017), and erythritol dehydrogenase *EYD1* (YALI0F01650g) (Carly et al., 2018). The striking feature of all these genes is their close localization on chromosome F. In this study we identified the role of another gene from this region, the gene *YALI0F01628*g. Moreover, qRT-PCR analysis showed the presence of more potential genes involved in erythritol assimilation in this yeast, suggesting a complex metabolic pathway of erythritol catabolism. Based on this and previous results we suggest a hypothetical pathway of erythritol assimilation in the yeast *Y. lipolytica*.

## Materials and Methods

### Strains

Strains used in this study are listed in **Table 1**. These strains belong to the Department of Biotechnology and Food Microbiology at Wrocław University of Environmental and Life Sciences, Poland.

**Table 1:** Strain list used in this study.

Strain	Genotype or plasmid	Source
***E. coli***		
DH5α	F^-^ endA1 glnV44 thi-1 recA1 relA1 gyrA96 deoR nupG Φ80dlacZΔM15 Δ(lacZYA-argF)U169, hsdR17(rK-mK+), λ-	Hanahan, 1985
DH5α	pAD, UAS1_B16_TEF promoter	Mirończuk et al., 2017
DH5α	pAD-EYI1, for overexpression of *YALI0F01628g*	This study
DH5α	pΔEYI1, for deletion of *YALI0F01628g*	This study
DH5 α	pΔku70, for deletion of Ylku70	This study
DH5 α	pΔku80, for deletion of Ylku80	This study
***Y. lipolytica***		
A101	Wild-type strain	Rzechonek et al., 2017
Wratislavia K1	Spontaneous mutant strain	Rymowicz et al., 2009
AJD	*MATA, A101*,Δura	Mirończuk et al., 2016
AJDD	*MATA*, AJD : ΔYlKU70, ΔYlKU780	This study
AJDD pAD-EYI1	*MATA*, AJDD, overexpression *YALI0F01628g*	This study
AJDD ΔEYI1	*MATA*, AJDD Δ *YALI0 F01628g*	This study
AJDD c-EYI1	*MATA*, AJDD complementation of *YALI0F01628*g	This study

### Media and Culture Conditions

*Escherichia coli* strains were cultivated in LB medium according to standard protocols. Rich yeast extract peptone glucose (YPD) medium, containing 1% (w/v) yeast extract, 1% (w/v) peptone, and 2% (w/v) glucose, was used to obtain yeast biomass for DNA extraction and inoculum preparation. Medium containing yeast nitrogen base (YNB) without amino acids (Sigma-Aldrich) supplied with 2% (w/v) glucose was used for yeast inoculum preparation.

During shake-flask experiments the cultures were grown in 0.3 L baffled flasks containing 0.03 L or 0.05 L medium on a rotary shaker (CERTOMAT IS, Sartorius Stedim Biotech) at 28°C and 240 rpm. Erythritol synthesis was conducted in Erythritol Synthesis Medium (ESM medium) containing 100 g/L glycerol (Chempur, Poland), 2.3 g/L (NH_4_)_2_SO_4_ (Chempur), 1 g/L MgSO_4_ × 7H_2_O (Chempur), 0.23 g/L KH_2_PO_4_ (Chempur), 26.4 g/L NaCl (Chempur), 1 g/L yeast extract (Merck, Germany), and 3 g/L CaCO_3_, pH 3.0. CaCO_3_ was added separately to each flask after establishing pH 3 in order to prevent a fall of pH value.

### Bioscreen C

The yeast strains were grown in 100-well plates in 150 μL of YNB supplemented with glucose 5% (w/v), erythritol 5% (w/v), or glycerol 5% (w/v). First, all strains were grown for 24 h in YNB medium containing 2% (w/v) glucose, next the strains were inoculated and grown for 48 h in YNB medium containing 2% (w/v) glucose, after it the strains were inoculated and grown for 72 h or in YNB medium containing 2% (w/v) glucose. The inoculum was grown two or three times on YNB medium to ensure that yeast cells did not accumulate nutrients. Finally the cells were inoculated to an OD_600_ value of 0.15 in each well. Quintuple experiments were performed at 28°C under constant agitation with a Bioscreen C system (Oy Growth Curves Ab Ltd., Finland). Growth was monitored by measuring the optical density at 420–560 nm every 30 min for 72 h.

### RNA Isolation and qRT-PCR

The shake flask cultures were grown for 24 h in YNB medium supplemented with a 5% (w/v) carbon source, i.e., glucose or erythritol. Next, the cultures were collected and centrifuged for 5 min at 12,000*g*. The RNA was extracted using the Total RNA Mini Plus kit (A&A Biotechnology, Poland). Each sample was treated with DNase I (Thermo Scientific) according to the manufacturer’s instructions. RNA quantities were measured using a Biochrom WPA Biowave II spectrophotometer (Biochrom Ltd., United Kingdom) equipped with a TrayCell (Hellma Analytics, Germany), and the samples were stored at -80°C. Synthesis of cDNA was conducted using Maxima First Strand cDNA. Synthesis kits for RT-qPCR (Thermo Scientific) were used according to the manufacturer’s instructions. qRT-PCR analyses were carried out using a DyNAmo Flash SYBR Green qPCR Kit (Thermo Scientific) and the Eco Real-Time PCR System (Illumina, United States).

Primers for qRT-PCR are listed in Supplementary Table 1. The results were normalized to the actin gene *YALI0D08272*g amplified with primers ACT-F/ACT-R and analyzed using the ddCT method (Schmittgen and Livak, 2008). Samples were analyzed in triplicate.

### Cloning and Transformation Protocols

All restriction enzymes were purchased from FastDigest Thermo Scientific and all of the digestions were performed according to standard protocols. The PCR reactions were set up using recommended conditions and Phusion high-fidelity DNA polymerase (Thermo Scientific). The ligation reactions were performed for 10 min at room temperature using T4 DNA Ligase (Thermo Scientific). The gel extractions were performed using the Gel Out extraction kit purchased from A&A Biotechnology (Poland). The *E. coli* minipreps were performed using the Plasmid Mini Kit (A&A Biotechnology). Transformation of *E. coli* strains was performed using standard chemical protocols.

Transformation was performed by the lithium acetate method and transformants were plated out on selective media without uracil as described before (Mirończuk et al., 2016). They were analyzed for proper integration by gDNA extraction and PCR amplification. Genomic DNA (gDNA) was extracted from *Y. lipolytica* using the Genomic Mini AX Yeast Spin kit (A&A Biotechnology, Poland).

### Construction of AJDD Strain

To increase homologous integration frequency in *Y. lipolytica*, in strain *Y. lipolytica* AJD we deleted genes encoding Ku70-Ku80 heterodimer that is known as an essential protein complex of the non-homologous end-joining (Kretzschmar et al., 2013). First, the 947 bp upstream region of *YALI0C08701g* (Ylku70) was amplified (primers Pku70-HindIII-F and Pku70-ApaI-R) and cloned into pUC_ura (Rzechonek et al., 2017) digested with HindIII and ApaI. Next, the obtained vector p_Pku70 was digested with SpeI and SacII and ligated with 1022 bp downstream region (amplified with primers Tku70-SpeI-F and Tku70-SacII-R) of YALI0C08701g, resulting in plasmid pΔku70. The obtained plasmid was digested with PmeI and HindIII create linear cassettes devoid of *E. coli* DNA. The transformants were plated out on selective media and were confirmed via gDNA extraction and PCR confirmations. Next, auxotrophy (*ura*-) was restored via excision using the Cre-lox recombinase as described before (Mirończuk et al., 2016).

Next, the upstream region of *YALI0E02068g* (Ylku80) was amplified with primers Pku80-HindIII-F and Pku80-ApaI-R), resulting in 1073 bp product and cloned into plasmid pUC_ura (Rzechonek et al., 2017) digested with HindIII and ApaI, yielding p_Pku80. The obtained vector was digested with SpeI and SacII and ligated with 1051 bp downstream region (amplified with primers Tku80-SpeI-F and Tku80-SacII-R) of *YALI0E02068g*, resulting in plasmid pΔku80. The obtained plasmid was digested with PmeI and HindIII create linear cassettes devoid of *E. coli* DNA. The transformants were plated out on selective media and proper integration in genome of strain named AJDD, were confirmed via gDNA extraction and PCR confirmations. Next, auxotrophy (*ura*-) was restored via excision using the Cre-lox recombinase as described before (Mirończuk et al., 2016).

### Construction of *YALI0F01628g* Deletion Cassette

First, the 350 bp downstream region of *YALI0F01628*g (primers DF01628-F and DF01628-R) was cloned to the plasmid pUC_ura (Rzechonek et al., 2017) digested with *BcuI* and *NotI*, yielding the pDown1628 vector. Next, the 350 bp *YALI0F01628*g upstream region was amplified by PCR using primers UPF01628-F and UPF01628-R and the PCR product was cloned into the pDown1628 vector digested with ApaI and PmlI, resulting in pΔEYI1. The obtained vector was digested with *HindIII* and *MssI* and used to transform *Y. lipolytica* AJDD, obtaining the *Y. lipolytica* AJDD Δ*EYI1* strain. Proper integration was verified by gDNA extraction and PCR analysis. Sequences of all primers used in the study are listed in Supplementary Table 1.

### Construction of Overexpression Cassette

The gene *YALI0F01628*g was amplified from *Y. lipolytica* DNA with primers F01628-SgsI-F and F01628-NheI-R, resulting in a 491 bp PCR fragment. It was digested with the enzymes *SgsI* and *NheI* and cloned into corresponding sites of plasmid pAD (Mirończuk et al., 2017), carrying the UAS1B_16_-TEF promoter. The obtained plasmid pAD-EYI1 was digested with *MssI* to create linear expression cassettes devoid of *E. coli* DNA and surrounded by *Y. lipolytica* rDNA for targeted integrations. It was used to transform *Y. lipolytica* AJDD or *Y. lipolytica* AJDD ΔEYI, resulting in strains AJDD pAD-EYI1 and AJDD c-EYI1, respectively.

### Bioreactor Studies

To prepare an inoculation culture for fermentation in a bioreactor, the cultures were grown in 0.3-L Erlenmeyer flasks (containing 0.1 L of YPD medium) on a shaker at 28°C for 72 h at 140 rpm. Erythritol production was conducted in a medium consisting of: 150 g/L glycerol, 2.3 g/L (NH_4_)_2_SO_4_, 1 g/L MgSO_4_ × 7H_2_O, 0.23 g/L KH_2_PO_4_, 26.4g/L NaCl, 1 g/L yeast extract, pH 3.0. An inoculum of 0.2 L was introduced into the bioreactor containing the production medium. The cultivations were performed in a 5-L jar bioreactor (Biostat B Plus, Sartorius, Germany) with a working volume of 2 L at 28°C. The aeration was fixed at 1.0 L/min. The stirrer speed was adjusted to 800 rpm. The pH was maintained automatically at 3.0 via the addition of NaOH (40% w/v). The amount of the supplied NaOH was taken into account during calculations of the metabolite concentrations. In order to limit evaporation during the batch cultures, the exhaust gases passed into the exhaust condenser in which the moisture was removed and returned to the vessel. The cultures were performed in three replicates.

### Analytical Methods

Samples (10 ml) from the cultures were centrifuged (10 min; 4°C; 5500 × *g*), harvested by filtration on 0.45-μm pore membranes and washed twice with distilled water. The biomass was determined gravimetrically after drying at 105°C. The concentration of polyols and citric acid in supernatant were determined with HPLC using a HyperRez Carbohydrate H^+^ Column (Thermo Scientific, Waltham, MA, United States) coupled to a UV (λ = 210 nm) (Dionex, Sunnyvale, CA, United States) and a refractive index (RI) detector (Shodex, Ogimachi, Japan). The column was eluted with 25 mM of trifluoroacetic acid (TFA) at 65°C and a flow rate of 0.6 ml min^-1^.

## Results

### *In Silico* Analysis of the Genes Responsible for Erythritol Utilization

**FIGURE 2:**
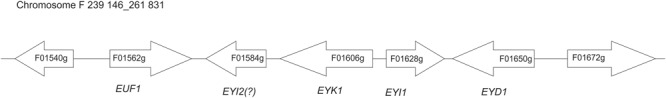
Localization of the gene in erythritol utilization cluster in *Y. lipolytica*.

Erythritol is an unusual carbon source, and the number of microorganisms that are able to assimilate it is limited. One of the best-studied microorganisms able to utilize erythritol is *Brucella abortus*, and its metabolic pathway of erythritol catabolism has been widely studied (Rodriguez et al., 2012). It was shown that in *Brucella*, erythritol utilization starts with phosphorylation by erythritol kinase (EryA) to yield L-erythritol-4-P, which is consequently oxidized by erythritol dehydrogenase (EryB) to L-3-tetrulose-4-P. Next, this compound is converted to D-erythrose-4-P via three isomerases: EryC, EryH (TpiA2), and EryI (RpiB). These genes are localized in two operons possessing two regulators (EryD and EryR) (Barbier et al., 2014; **Figure 1**). In *Y. lipolytica* the same enzymes had been found, the sequence similarity between these two species is low (**Table 2**). Proteins EryA and EryB showed low identity to *YALI0F00484p* (24%) and *YALI0F13970p* (33%), which are glycerol kinase and glycerol mitochondrial dehydrogenase, respectively. Homologs for EryC, EryD, and EryR proteins cannot be found in *Y. lipolytica*. Interestingly, two isomerases described in *Brucella abortus* have homologs in *Y. lipolytica*. The protein EryI shows high similarity to the protein encoded by the gene *YALI0F01628g* (47%). Moreover, EryH shows 37% similarity to the protein YALI0F1584p. Both of the genes are localized in the same region as previously described EUF1, EYK1, and EYD1 (**Figure 2**).

**Table 2:** Comparison of the proteins in erythritol pathway from *Brucella abortus* and *Yarrowia lipolytica.*

Protein in *B. abortus*	Protein in *Y*. *lipolytica*	Identity [%]	Query cover [%]	Function in *B. abortus*	Putative function based on the proteins’ domain in *Y. lipolytica*
EryA (BAB2_0372)	YALI0F00484p	24%	88%	Erythritol kinase	Glycerol kinase
EryB (BAB2_0371)	YALI0B13970p	32%	89%	Glycerol-3-phosphate dehydrogenase	Glycerol-3-phosphate dehydrogenase mitochondrial
EryC (BAB2_0370)	–	–	–	Tetrulose- 4-phosphate racemase	–
EryD (BAB2_0369)	–	–	–	DNA-binding transcriptional regulator	–
EryR (BAB2_0368)	–	–	–	DeoR family transcriptional regulator	–
EryH (BAB2_0367)	YALI0F01584p	37%	78%	D-3-tetrulose-4-P isomerase	Triose-phosphate isomerase
EryI (BAB2_0366)	YALI0F01628p	48%	70%	D-erythrose -4-P isomerase	Ribose-5-phosphate isomerase

### Genes From the Erythritol Utilization Cluster Are Highly Expressed in the Presence of Erythritol

**FIGURE 3:**
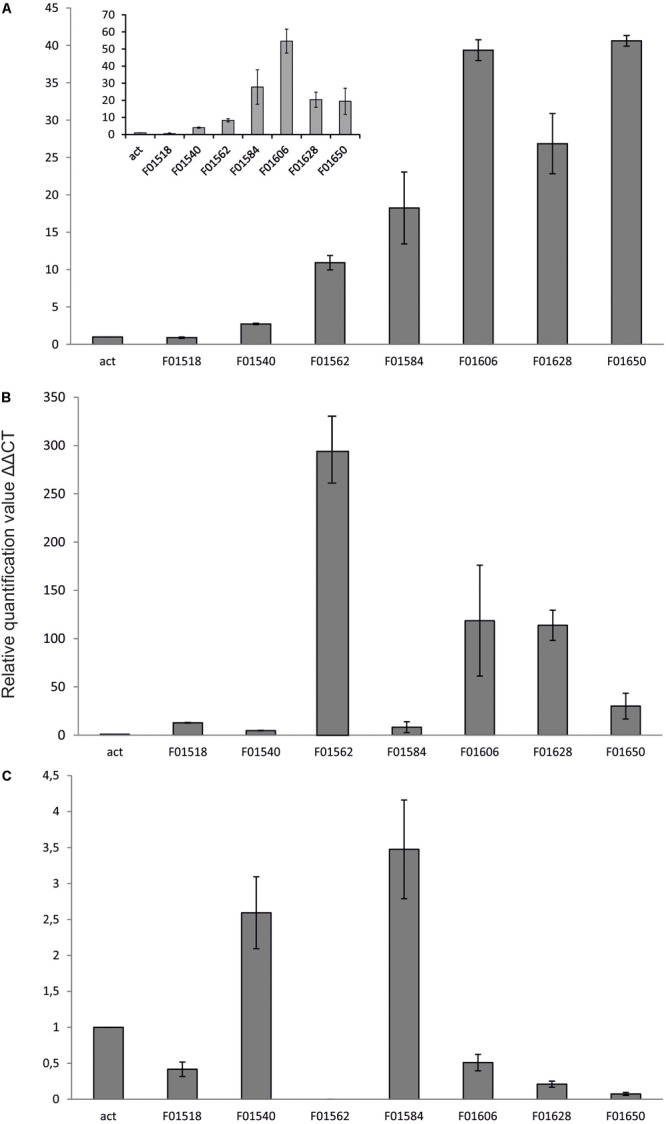
Expression of genes, **(A)** *Y. lipolytica* A101 on medium with glucose and erythritol as a sole carbon source. Inset: expression of the same genes in the presence of erythritol but in strain AJDD. Strain *Y. lipolytica* AJDD was grown on medium with erythritol as a sole carbon source or on medium with glucose (the control). **(B)** *Y. lipolytica* overexpressing EUF1 on medium with glucose. **(C)** *Y. lipolytica* with deleted EUF1 on medium with glucose. For **B,C** wild-type A101 was used as a reference strain. Samples were analyzed in triplicate, and the standard errors were estimated using Illumina Eco software.

Yeast *Y. lipolytica* produces erythritol under high osmotic pressure to protect the cells against outflow of water. Production of erythritol by this yeast has been widely studied (Rymowicz et al., 2009; Yang et al., 2014; Rakicka et al., 2017b), but the assimilation pathway of erythritol remains a puzzle. Despite the fact that this yeast can assimilate this polyol as a carbon source, some of the wild-type strains cannot utilize this compound. Genome mining led us to find an erythritol utilization factor (*EUF1*), described before (Rzechonek et al., 2017). *EUF1* is localized on chromosome F (gene number *YALI0F01562*g), surrounded by genes encoding putative erythrulose kinase (*EYK1, YALI0F01606g*) (Carly et al., 2017) and erythritol dehydrogenase (*EYD1, YALI0F01650g*) (Carly et al., 2018). Thus, the first step in our study was examination of the expression level of the genes localized on chromosome F close to *EUF1* in the region named the “erythritol utilization cluster” (**Figure 1**). To this end, we compared the relative expression of the genes from the cluster, under two different conditions. First, the strain *Y. lipolytica* A101 was grown in YNB medium supplemented with erythritol. Second, strain A101 was grown in YNB medium supplemented with glucose. Remarkably, we noted that five of the seven selected genes showed significantly higher expression in the presence of erythritol than in the control (**Figure 3A**). Similar results were obtained when strain AJDD was grown in YNB medium supplemented with erythritol vs. glucose (**Figure 3A**, inset). Three of them had been already described, but two of them, *YALI0F01584g* and *YALI0F1628g*, have never been described as genes involved in erythritol assimilation. The first gene belongs to the triosephosphate isomerase superfamily (tpiA2) and the latter is putatively ribose/galactose isomerase type B (rpiB). Because in previous research we had found the transcriptional factor *EUF1*, we examined the effect of its overexpression on the expression level of genes from the cluster (**Figure 3B**). To eliminate the influence of erythritol, the strains (wild type and *EUF1* overexpressing strain) were grown in YNB medium supplemented with glucose. We noted that almost all genes from the cluster showed elevated expression, whereas the opposite effect was observed in the strain with deleted *EUF1*. Genes encoding *EYK1, EYD1*, and *YALI0F1628g* showed significantly lower expression than the wild type (**Figure 3C**). Interestingly, we observed that the genes *YALI0F1628g, EYK1*, and *EYD1*, were highly repressed in the presence of glycerol. Expression level of *EUF1* gene remained the same as in the control medium. We did not observe any difference in the gene expression level between wild-type A101 and the modified strain AJDD (ΔYlKu70 and ΔYlku80) (Supplementary Material). Because the gene *YALI0F1628g* showed stronger dependency on *EUF1*, we chose it for further study.

### Deletion of *YALI0F1628*g Results in Impairment of Erythritol Assimilation

According to the Genome Resources for Yeast Chromosomes (GRYC) website, the protein encoded by the *YALI0F1628g* gene is a putative ribose-5-phosphate isomerase. The proteins which belong to the family are responsible for conversion of ribulose-5-P to ribose-5-phosphate in the PPP. Next, we aimed to modify the *Y. lipolytica* AJDD strain to delete (AJDD ΔEYI1), overexpress (AJDD pAD-EYI1), and complement (AJDD c-EYI1) the *YALI0F01628g* gene to verify its role in erythritol assimilation.

**FIGURE 4:**
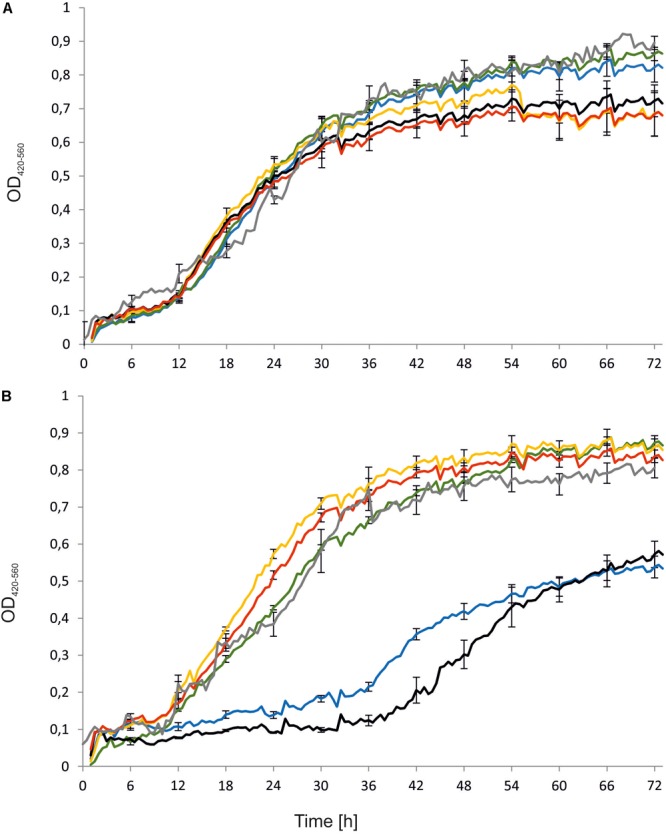
Growth curves of *Y. lipolytica* strains: A101 (green), AJDD (gray), Wratislavia K1 (blue), AJDD pAD-EYI1 (yellow), AJDD ΔEYI1 (black), and AJDD c-EYI1 (red). Strains were grown on YNB medium supplemented with 5% glucose **(A)** or 5% erythritol **(B)** as a sole carbon source. Experiment was performed at 28°C under constant agitation using Bioscreen C. The standard deviations were <10% of the values shown.

First, the engineered strains, namely *Y. lipolytica* AJDD ΔEYI1, AJDD pAD-EYI1, and AJDD c-EYI1, were tested for their ability to grow on different carbon sources, i.e., erythritol and glucose. As a control the parental strain (AJDD) and the wild-type (A101) were used. In addition, as a negative control we used the strain *Y. lipolytica* Wratislavia K1, the wild-type strain which possesses a natural defect for erythritol assimilation (Rzechonek et al., 2017). We did not observe any substantial difference in growth on glucose nor on erythritol for A101 and AJDD strain, thus A101 was used in next experiments as a control strain. An interesting result was observed in the medium with erythritol as a carbon source (**Figure 4**). The strain with a deleted *YALI0F01628g* gene was unable to grow on erythritol for about 40 h. Its growth was significantly delayed in comparison to the parental strain A101 and it grew much more weakly than the strain Wratislavia K1 (the negative control). Interestingly, after this period the modified strain (AJDD ΔEYI1) started the log phase of growth. The opposite effect was observed for the strain bearing an overexpression cassette (AJDD pAD-EYI1). This strain obtained the highest OD_600_, already after 12 h. Of note, the gene *YALI0F01628g* was cloned under the UAS1B_16_-TEF promoter, which shows the highest activity after 24 h (Blazeck et al., 2013). Indeed, after this period, the growth of AJDD pAD-EYI was the highest, and it was improved in comparison to strain A101 and AJDD. The complementation strain AJDD c-EYI1 showed better growth than strain A101, but it achieved a lower OD_600_ value than AJDD pAD-EYI1. This might be caused by an additional copy of *YALI0F01628g* in the native locus in strain AJDD pAD-EYI1.

### The Gene *YALI0F01628*g Is Involved in Erythritol Catabolism

**FIGURE 5:**
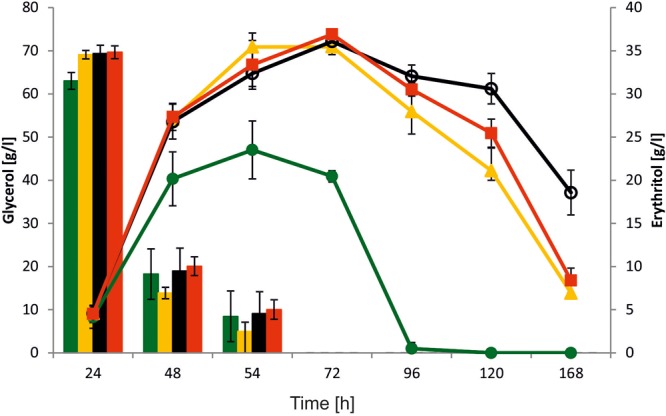
Shake-flask experiment with various *Y. lipolytica* strains: A101 (green), AJDD pAD-EYI1 (yellow), AJDD ΔEYI1 (black), and AJDD c-EYI1 (red). 100 g/L glycerol assimilation (bars) and erythritol production/assimilation (lines). The cultures were performed in three biological replicates. Error bars represent standard deviation.

Yeast *Y. lipolytica* is known as a good host for erythritol production. Effective production of this polyol is induced by high osmotic pressure, mainly at low pH because of inhibition of organic acid synthesis. To explore the role of *YALI0F1628g* in erythritol synthesis, we performed a shake-flasks experiment with the modified strain and the wild-type strain as a control. To this end, strains were grown in baffled flasks with Erythritol Synthesis Medium (Mirończuk et al., 2016) to induce a natural process of erythritol biosynthesis. In this study we used glycerol as a carbon source, since it has a greater impact on erythritol synthesis than glucose. Glycerol is the principal a side-product deriving from biodiesel facilities, and besides the conversions realized by *Y. lipolytica*, several value-added compounds can be generated through microbial conversion of this renewable material (Lindlbauer et al., 2017; Papanikolaou et al., 2017a). As seen in **Figure 5**, all tested strains showed a different pattern of erythritol synthesis. Surprisingly, all modified strains showed improved erythritol synthesis in comparison to the wild-type strain. The glycerol uptake and assimilation were similar among all tested strains and all of them utilized 100 g/L of substrate within 72 h. When the main carbon source was depleted, the control strain started to utilize erythritol and the other metabolites such as mannitol, arabitol, and citric acid (Supplementary Material). The erythritol was utilized within the next 24 h. Surprisingly, we did not observe any difference between strains AJDD ΔEYI1, AJDD pAD-EYI1, and AJDD c-EYI1 for the first 72 h of cultivation. All of them produced a similar amount of erythritol (≈36.0 g/l). However, when the main carbon source was depleted, differences between strains occurred. Strain AJDD ΔEYI1 was almost unable to utilize erythritol for the next 48 h; the difference in the amount of this polyol at 72 and 120 h of cultivation was 36.9 and 30.9 g/l, respectively. The strain with overexpression of *YALI0F1628*g started to utilize erythritol rapidly and within 48 h its amount decreased by 40%. The strain with complementation was able to consume erythritol sooner than the deletion strain but slightly slower than the strain bearing an overexpression cassette. Interestingly, we noted that all modified strains produced more citric acid than the wild-type strain (Supplementary Material). The metabolite was produced during the first 72 h of cultivation, after which it became a carbon source for the yeast. Interestingly, the wild-type *Y. lipolytica* A101 produces significantly more biomass (12 g/L), than all engineered strains (8–9 g/L). Thus in WT, carbon flux was strongly directed into biomass production but not into erythritol synthesis. This explains differences between the strains in erythritol titer. For industrial application, the best erythritol producer among the tested strains was strain AJDD ΔEYI. This strain is able to produce high quantities of erythritol and its erythritol assimilation was impaired. For this reason this strain was chosen for further studies.

### Fermentation Performance of the AJDD ΔEYI Strain

**FIGURE 6:**
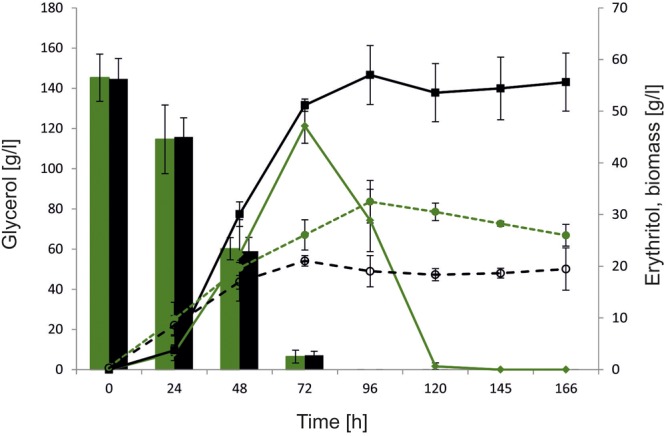
Bioreactor experiment with *Y. lipolytica* strains A101 (green) and AJDD ΔEYI1 (black), 150 g/L glycerol assimilation (bars) and erythritol production/assimilation (lines) and biomass synthesis (dashed lines). The cultures were performed in three biological replicates. Error bars represent standard deviation.

To further characterize the role of *YALI0F01628*g and explore its influence on erythritol synthesis and assimilation, large-scale fermentation was conducted using a 5-L stirred-tank bioreactor. Glycerol concentration was increased to 150 g/l to promote erythritol biosynthesis by enhanced osmotic pressure. In this study we used as a control the wild-type strain A101. To verify the effect of *YALI0F01628*g deletion on erythritol catabolism, the fermentation was extended to 168 h, which allowed us to monitor the erythritol assimilation after depletion of the main carbon source. Similarly to the previous experiment, we did not observe any significant difference in glycerol assimilation between the tested strains. Both the control strain and AJDD ΔEYI1 utilized 150 g/L of glycerol within 96 h. As seen in **Figure 6**, also in large-scale fermentation, strain AJDD ΔEYI1 produced more erythritol than the control. The modified strain produced 57 g/l of erythritol and the control strain only 47 g/L. For first 24 h, the amount of the synthesized erythritol was at a similar level for both strains. However, the modified strain continued the production of polyol until 96 h of fermentation, whereas the control strain started to utilize erythritol immediately after glycerol depletion. Moreover, the strain with the deleted *YALI0F01628*g gene was almost unable to assimilate erythritol for the next 70 h after glycerol depletion. In contrast to the control, it utilized synthesized citric acid, instead of erythritol and other polyols (Supplementary Material). The next important difference between strains was biomass production. The control strain produced 35% more biomass (g/l) than strain AJDD ΔEYI1. It is clear that the assimilated carbon was redirected into synthesis of biomass, instead of synthesis of the metabolites. Moreover, cell proliferation was inhibited and the biomass of the control strain A101 decreased after 96 h of fermentation. An opposite effect was observed for the modified strain: its biomass remained almost unchanged during the stationary phase of growth. These data clearly showed that strain AJDD ΔEYI1 is an efficient producer of erythritol, and the deletion of *EYI* does not have an negative effect on erythritol biosynthesis or on the cell condition.

## Discussion

The identification of a new gene involved in erythritol catabolism is an important step in understanding the physiology and the underlying regulatory mechanism of erythritol assimilation in the yeast *Y. lipolytica*. Erythritol synthesis in *Y. lipolytica* has been partially described (Mirończuk et al., 2017), but some important information is still missing. In our previous study, we identified in *Y. lipolytica* a new transcriptional factor, *EUF1* (Rzechonek et al., 2017), suggesting the presence of an unknown metabolic pathway involved in erythritol assimilation. Thus we started to study this phenomenon in detail. Recently two important genes encoding erythrulose kinase (Carly et al., 2017) and erythritol dehydrogenase have been identified (Carly et al., 2018). All of these genes are localized on chromosome F at a small distance from each other; thus we named this region the erythritol utilization cluster. In prokaryotes, genes of a common function are often organized in operons. Although most eukaryotes show much weaker clustering of genes by function, gene clustering does occur in eukaryotes (Lee and Sonnhammer, 2003; Thevenin et al., 2014). This led us to verify the expression level of the genes in the presence of erythritol to find out if more genes from the cluster are involved in erythritol assimilation. As mentioned above, most of the selected genes showed an elevated expression level. However, what is more interesting, most of the genes responded positively to *EUF1* overexpression, showing enhanced expression in medium supplemented with glucose as a carbon source. Here we discovered that *EUF1* might be responsible for activation of the genes in the erythritol utilization cluster. The result was confirmed in the experiment with the strain with the deleted *EUF1* gene: most of the genes localized in the cluster showed repressed gene expression (**Figure 3**).

To confirm the role of *YALI0F01628g* in erythritol catabolism, we made strains with deletion (AJDD ΔEYI1), overexpression (AJDD pAD-EYI1), and complementation (AJD c-EYI1) of the gene. On note, the strain with deleted *YALI0F01628g* was unable to grow on erythritol for the first 36–40 h of incubation. After this period, the strain started to grow and we observed a slow but continuous increase of OD_600_. Previously, a similar effect was observed for *Y. lipolytica* with deletion of the *EUF1* gene; after an extended lag phase the stain was able to grow on erythritol as a sole carbon source (Rzechonek et al., 2017). Unfortunately, we can only presume that the strains with deleted *EYK1* and *EYD1* genes also showed impaired growth on erythritol after a long lag phase. The authors reported only 8–12 h of growth on medium with erythritol, but in the fermenter studies the strain *Y. lipolytica* RIY208 (Δeyk1) showed a decrease in erythritol level after 48 h of fermentation (Carly et al., 2018). All these results suggest that in *Y. lipolytica* there might exist at least two metabolic pathways of erythritol catabolism – a major and secondary pathway of erythritol assimilation. When the major pathway is disturbed at some level, the secondary pathway is activated by an unknown factor. It allows for erythritol assimilation independently from the major pathway. Here, we want to suggest the possibility that genes from the erythritol utilization cluster are involved in the major pathway of erythritol assimilation.

Puzzling results were obtained in the shake-flask experiments, because both deletion and overexpression strains showed elevated erythritol synthesis in comparison to the control. Of note, the erythritol level did not differ between AJDD ΔEYI and AJDD pAD-EYI1 strains for the first 72 h of cultivation. It was observed that glycerol represses the expression of the genes from erythritol utilization cluster, with exception for *YALI0F01584*g and *EUF1* (Supplementary Material). Thus it explains why strain AJDD pAD-EYI1 did not utilize the synthetized polyol. After depletion of glycerol, the differences between the strains occurred. Interestingly, the strain with deleted *YALI0F01628*g needed more than 24 h to start efficiently utilizing erythritol, confirming the results from the previous experiment. Strain AJDD pAD-EYI1 rapidly started to assimilate this polyol. This result implies that the *YALI0F01628*g gene is not essential for erythritol synthesis; on the contrary, the major pathway of erythritol assimilation can be inverted and redirected for erythritol synthesis. At the beginning of the fermentation high concentration of glycerol represses the pathway of erythritol utilization. The overexpression of *YALI0F01628*g leads to enhanced production of erythritol, because it is redirected into erythritol synthesis. The environmental conditions force the cells into enhanced erythritol synthesis; thus strain AJDD pAD-EYI1 produced more erythritol than the control. When the glycerol was depleted, erythritol became the main carbon source and its high concentration induced the erythritol utilization cluster. The strain overexpressing *YALI0F01628g* assimilated erythritol sooner than the other modified strains. In the contrary, to the other studies where high co-production mannitol and erythritol was observed (Papanikolaou et al., 2017b), in this study low production of the other polyols was observed (Supplementary Material). Moreover, the wild-type strain A101 produced almost 50% more biomass (12 g/L), than the engineered strains (8–9 g/L). This data explains the lowest titer of erythritol and the fact that the wild-type showed the higher rate of the erythritol assimilation in later stages of the cultivation among the tested strains.

In agreement with the shake-flask experiment, strain AJDD ΔEYI1 produced more erythritol than the control strain in the bioreactor study. The erythritol titer was significantly higher and production time was shorter than in the other studies where the strain with disrupted erythritol catabolism was used. In this study strain AJDD ΔEYI1 produced 57 g/L of erythritol within 96 h, while the strain with ΔEYK1 produced 35 g/L of erythritol during 180 h of cultivation (Carly et al., 2017). Moreover, carbon from glycerol is redirected into erythritol synthesis, and not into biomass synthesis. Production of biomass by AJDD ΔEYI1 strain was significantly lower than for the wild-type (**Figure 6**). Similar level of biomass production from glycerol was observed before (Papanikolaou et al., 2017b). These data imply that strain AJDD ΔEYI1 is a promising producer of erythritol on the industrial scale and it is a good platform for further modification.

The role of *YALI0F01628g* in erythritol synthesis is not crucial, suggesting that the metabolic pathways of erythritol anabolism and catabolism are separate.

An interesting effect of the dual (catabolism and anabolism) function of the major pathway of erythritol assimilation was observed before. In *Y*. *lipolytica* strain FCY221 (Δeyk1, pTEF-GUT1-TKL1) overexpressing *EYD1* the erythritol productivity was 44% lower than in its parental strain (Carly et al., 2018). Despite the fact that EYD1 was overexpressed, the authors did not observe erythrulose (intermediate product) synthesis, which was the main aim of the experiment. In the same strain transketolase (*TKL1*) was overexpressed. It is necessary to point out that *TKL1* is involved in the PPP and it was shown as a crucial gene for erythritol synthesis (Mirończuk et al., 2017). Thus under osmotic stress overexpression of *TKL1* results in redirection of carbon flux for the major pathway of erythritol synthesis, including in the final step reduction of erythrose to erythritol by erythrose reductase (YlER) with NADH as a cofactor (Janek et al., 2017). Simultaneous overexpression of *EYD1* and *TKL1* in the Δeyk1 strain results in disruption of the carbon flux since the secondary pathway of erythritol synthesis was blocked. This might result in lower erythritol synthesis.

**FIGURE 7:**
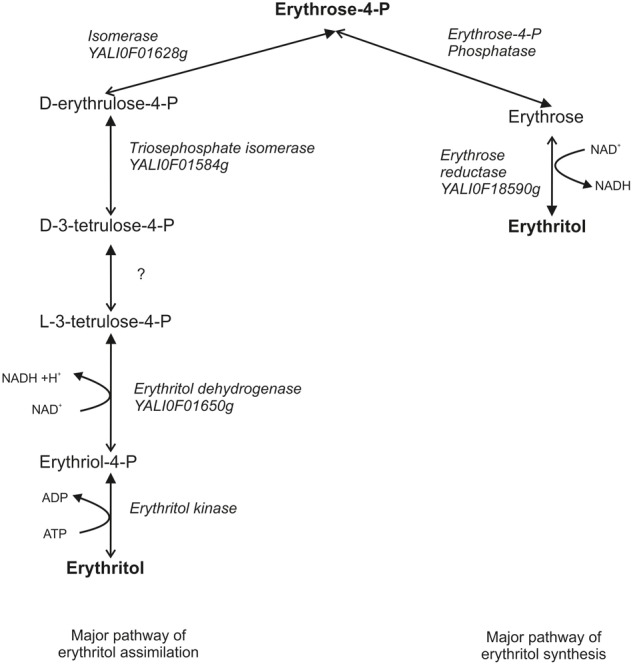
Hypothetical pathway of erythritol metabolisms in *Y. lipolytica*. Closed arrows indicate the major role of the pathway. Opened arrows indicate the secondary role of pathway.

At this point we would like to suggest a new hypothetical metabolic pathway of erythritol assimilation. We presume that by analogy to glycerol assimilation in *Y. lipolytica* and the erythritol catabolism pathway in *Brucella abortus*, first erythritol is phosphorylated by erythritol kinase to L-erythritol-4-P, which is next oxidized by the dehydrogenase EYD1 to L-3-tetrulose-4-P. Consequently, L-3-tetrulose-4-P is converted to D-erythrose-4-P via isomerases YALI0F01584p and YALI0F1628p. D-erythrose-4-P is incorporated into PPP and consequently to glycolysis/TCA (**Figure 7**). This hypothesis is supported by the fact that dihydroxyacetone kinase is found in bacterial genomes devoid of eryA and eryB. Homologs of the isomerases are involved in the metabolisms of L-erythrulose, which is phosphorylated by dihydroxyacetone kinase to D-3-tetrulose-4P and isomerized to D-erythrose-4-P (Barbier et al., 2014). *EYK1* was described as a protein with (100%) homology to the putative dihydroxyacetone kinase (Carly et al., 2017). Because synthesis of erythritol occurs via PPP, the results obtained in this study lead us to suggest that both catabolism and anabolism metabolic pathways of erythritol might be connected by D-erythrose-4-P and both of them might be redirected if necessary. Production of erythritol is crucial for *Y. lipolytica* to survive under high osmotic pressure, which might explain the presence of two metabolic pathways of erythritol production. On the other hand, erythritol is an atypical carbon source, and it can be reused when other carbon sources are depleted. While this hypothesis needs further studies to be proven, our findings provide new insights into erythritol metabolism in the yeast *Y. lipolytica*.

## Author Contributions

AM designed the study, constructed the deletion and overexpression cassette, performed the qRT-PCR analysis, and wrote the manuscript. AB performed the shake-flask experiments and bioreactor experiments. KZ performed the Bioscreen C experiment. DR and AD revised the manuscript.

## Conflict of Interest Statement

The authors declare that the research was conducted in the absence of any commercial or financial relationships that could be construed as a potential conflict of interest.
