# Supplementary material for: A Role of a Newly Identified Isomerase From Yarrowia lipolytica in Erythritol Catabolism
Source: Front Microbiol. 2018 May 30;9:1122. doi: 10.3389/fmicb.2018.01122 (PMC5992420; doi:10.3389/fmicb.2018.01122)
Supplement: Supplementary file 1 [file Data_Sheet_1.docx]

Supplementary Material

**A role of a newly identified isomerase from *Yarrowia lipolytica* in erythritol catabolism**

**Aleksandra M. Mirończuk*, Anna Biegalska, Karolina Zugaj, Dorota A. Rzechonek, Adam Dobrowolski**

Department of Biotechnology and Food Microbiology, Wroclaw University of Environmental and Life Sciences, Chełmońskiego 37, 51-630, Wrocław, Poland

*corresponding author


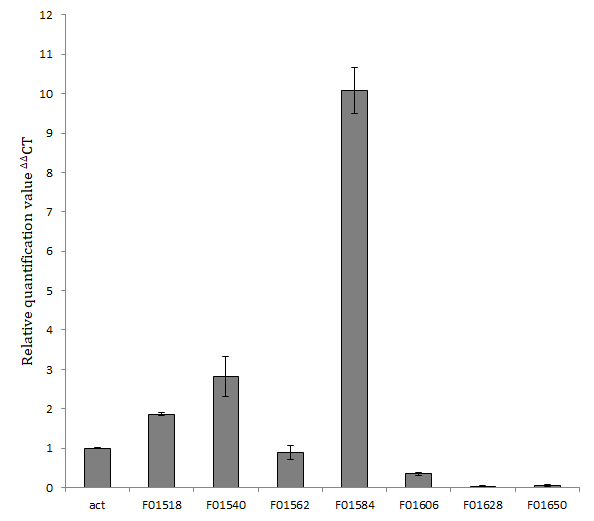


**Supplementary Figure 1**. Expression of genes in this study in the presence of glycerol. Relative quantification of RNA transcript using RT-PCR; actin was used as a reference gene. Strain *Y. lipolytica* A101 was grown on medium with glycerol as a sole carbon source or on medium with glucose (the control). Samples were analyzed in triplicate, and the standard errors were estimated using Illumina Eco software.


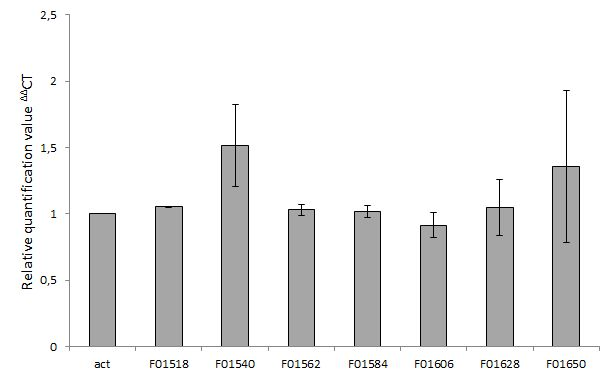


**Supplementary Figure 2**. Expression of genes in this study in two strains of *Yarrowia lipolytica* A101 and AJDD. Relative quantification of RNA transcript using RT-PCR; actin was used as a reference gene. Strains *Y. lipolytica* A101 and AJDD were grown on medium with glucose as a sole carbon source. Samples were analyzed in triplicate, and the standard errors were estimated using Illumina Eco software.


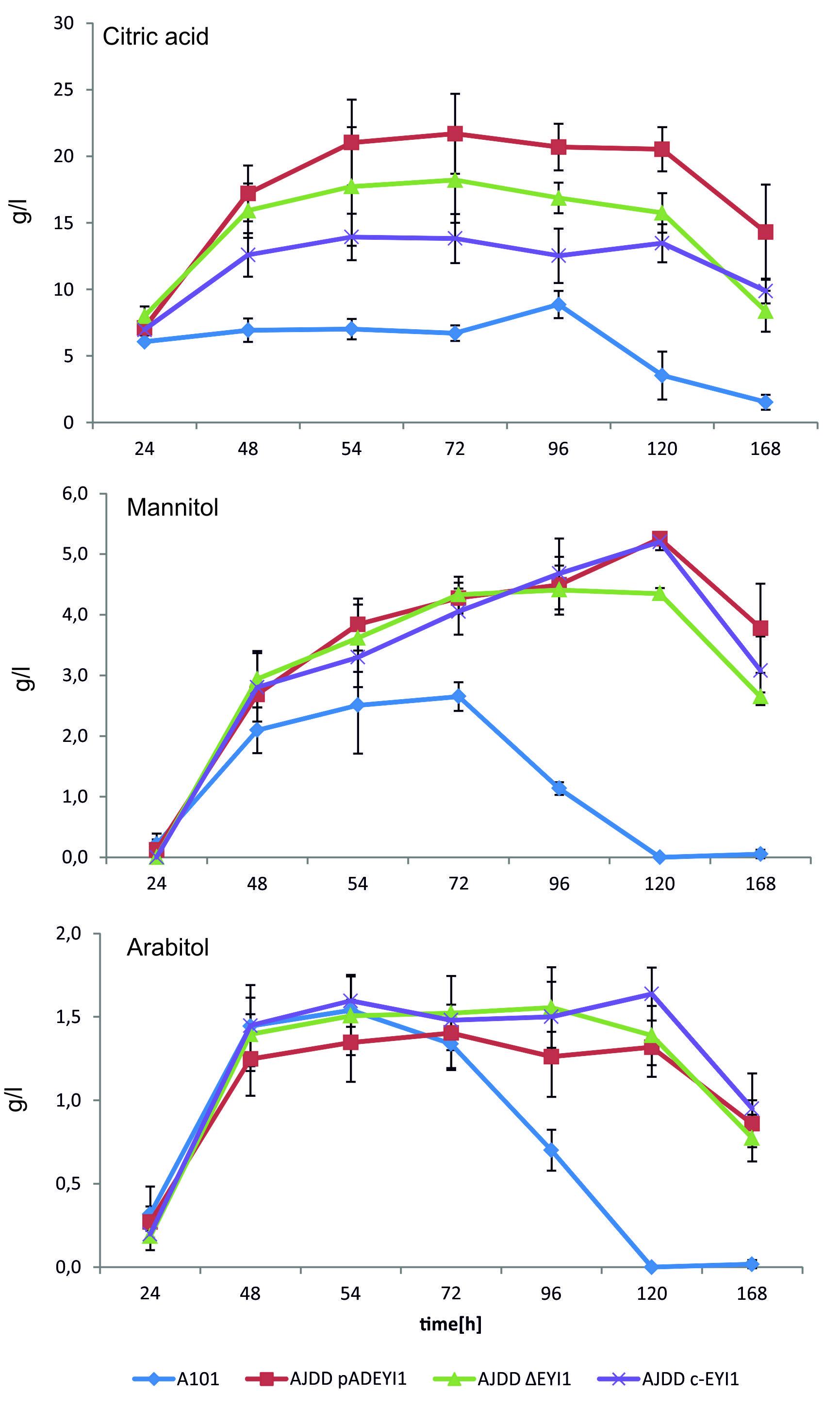


**Supplementary Figure 3.** Production of the side-metabolites by various strain of *Y. lipolytica* in shake-flask experiment.


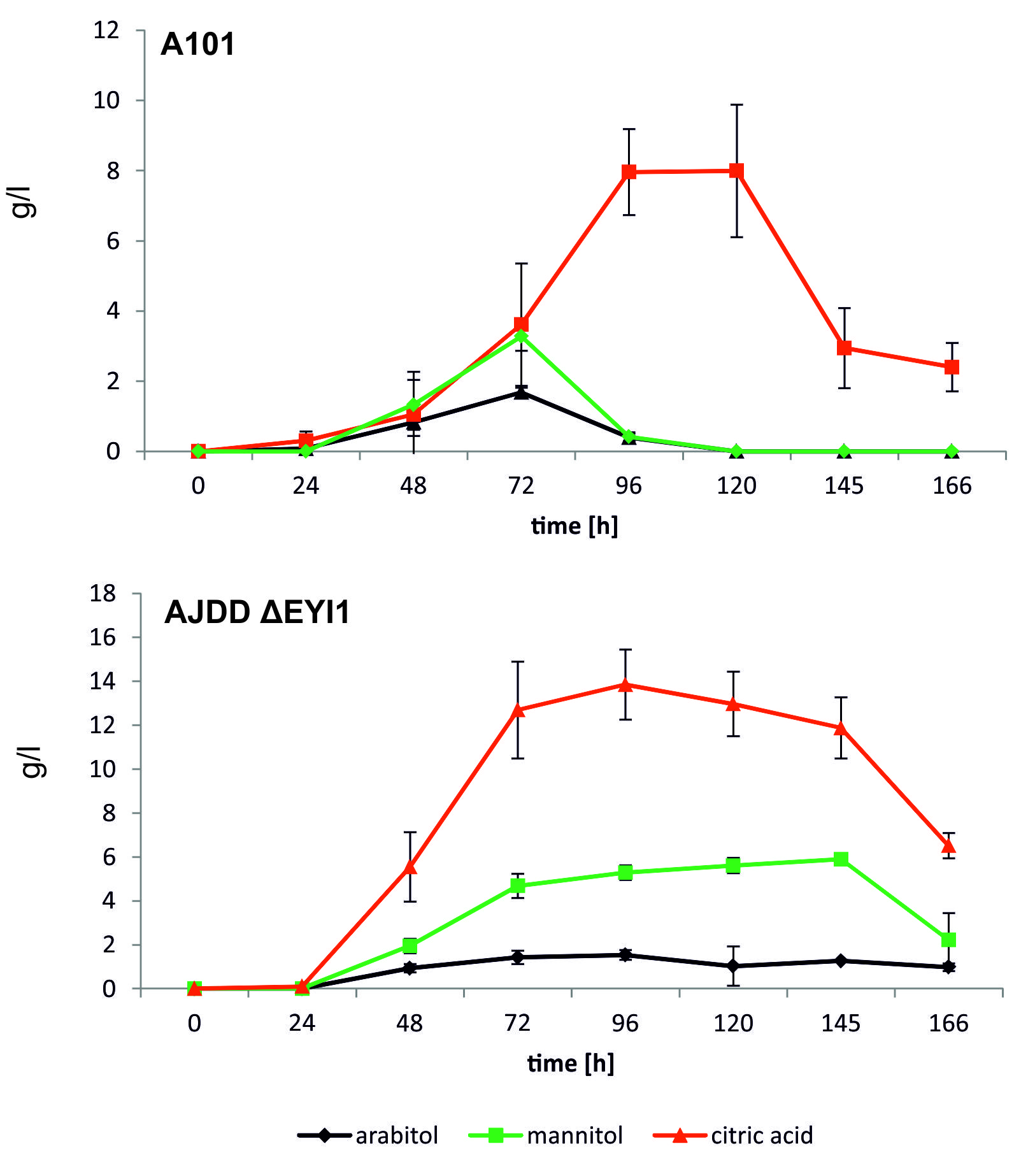


**Supplementary Figure 4.** Production of side-metabolites by strain of *Y. lipolytica* during bioreactor study.

**Supplementary Table 1**. Primer list used in this study.

| **Name** | **Sequence 5’-3’** |
| --- | --- |
| UPF01628-F | CCTCACGTGCGTTGACCCCCACGCTGT |
| UPF01628-R | TAAGGGCCCAATCCCATTTTGTGCAAGTG |
| DF01628-F | CGCACTAGTCACATTGCTGCCTAAACG |
| DF01628-R | TAAGCGGCCGCGGAGAACAGGAGAATGG |
| Tku80-SpeI- | CGCACTAGTCTGGTTGCACAGATCAACTC |
| Tku80-SacII-R | TAACCGCGGCACTTACGCCAAGACC |
| Pku80-HindIII-F | CATAAGCTTCTCGATCCGGACAAGG |
| Pku80-ApaI-R | TACGGGCCCATGCCATGCTCAAAGTC |
| Tku70-SpeI-F | CGCACTAGTTGACTAGGGAGGCACATC |
| Tku70-SacII-R | TACCCGCGGAAGTGAACGACCAAGAC |
| Pku70-HindIII-F | CGCAAGCTTCGACAGCACTCGTACTC |
| Pku70-ApaI-R | TACGGGCCCTTCGTGGTTCGTGTTTC |
| qF01650-F | AGCCGCTACTTCTGCTCTGC |
| qF01650-R | TCCAGAGCAGCATTGAGTCC |
| qF01628-F | ACGAGGCTGGCGTTGATTAC |
| qF01628-R | CCTTGACACCAACGTCAATG |
| qF01606-F | AGGGAGTTCAGGCTTCCCGATCAG |
| qF01606-R | CCAGAGCCTCCTCCAGAAAG |
| qF01584-F | TACTTTGATCCGGCCAAGAC |
| qF01584-R | CTGGACGGGTCCAGTGATTC |
| qF01562-F | ACCGGAGACGTCCAGAGTTC |
| qF01562-R | TATCGGTGCCCTTCTTGGTG |
| qF01540-F | GTTCTGTCGGGCGAATTTCC |
| qF01540-R | ATGGCCAGTTTCAGGTCATC |
| qF01518-F | TCCCTGGTTGGATCAATGAC |
| qF01518-R | GGAAGGACCGGGCTTATCAG |
| Act-F | GAGTCACCGGTATCGTTC |
| Act-R | GCGGAGTTGGTGAAAGAG |
